# Multi-scale crystallographic ordering in the cold-water coral *Lophelia pertusa*

**DOI:** 10.1038/s41598-017-09344-5

**Published:** 2017-08-21

**Authors:** Vincent Mouchi, Pierre Vonlanthen, Eric P. Verrecchia, Quentin G. Crowley

**Affiliations:** 1 Sorbonne Universités, UPMC Univ. Paris 06, CNRS UMR 7193, ISTEP, F-75005 Paris, France; 2 Geology, School of Natural Sciences, Trinity College, Dublin, Ireland; 3 Institute of Earth Sciences, FGSE, Géopolis, University of Lausanne, Lausanne, Switzerland; 4 Institute of Earth Surface Dynamics, FGSE, Géopolis, University of Lausanne, Lausanne, Switzerland

## Abstract

*Lophelia pertusa* is a widespread colonial cold-water coral which can form large three-dimensional habitats for benthic communities. Although it is known to construct an aragonite skeleton with optically opaque and translucent bands, details of its biomineralized structure are unclear. New crystallographic data obtained from *Lophelia pertusa* using electron backscatter diffraction (EBSD) reveal a remarkably high degree of multiscale self-ordering and provide unprecedented detail on crystallographic orientations within the coral skeleton. The EBSD data unequivocally demonstrate a self-regulated architecture across a range of spatial scales, resulting in a specific structure which contributes to the physical robustness of its skeleton and an evolutionary advantage in such habitats.

## Introduction

*Lophelia pertusa* is a colonial reef-building scleractinian cold-water coral species widely distributed in Plio-Pleistocene to modern marine waters^1^. It dominates modern reef frameworks in the north-east Atlantic and is found in several cold-water marine ecosystems worldwide (e.g. Pacific and Indian oceans)^2^. Contemporary cold-water corals can form large carbonate mounds^3^, which create significant three-dimensional sea-floor habitats^1, 4^ often resulting in biodiversity hot-spots, particularly as a nursery for juvenile fish species^5^. Cumulatively, cold-water corals also constitute an important but poorly-quantified carbon sink in the oceans^6^. Whereas the biochemical and metabolic effects of rising atmospheric CO_2_ concentrations and ocean acidification on cold-water corals are not fully understood^7^, Hennige *et al*.^8^ reported a decrease in breaking strength of *L. pertusa* under such conditions. Given that *L. pertusa* typically resides in areas of intensified seabed currents^4^, a structurally robust skeleton is essential for its survival.

**Figure 1:**
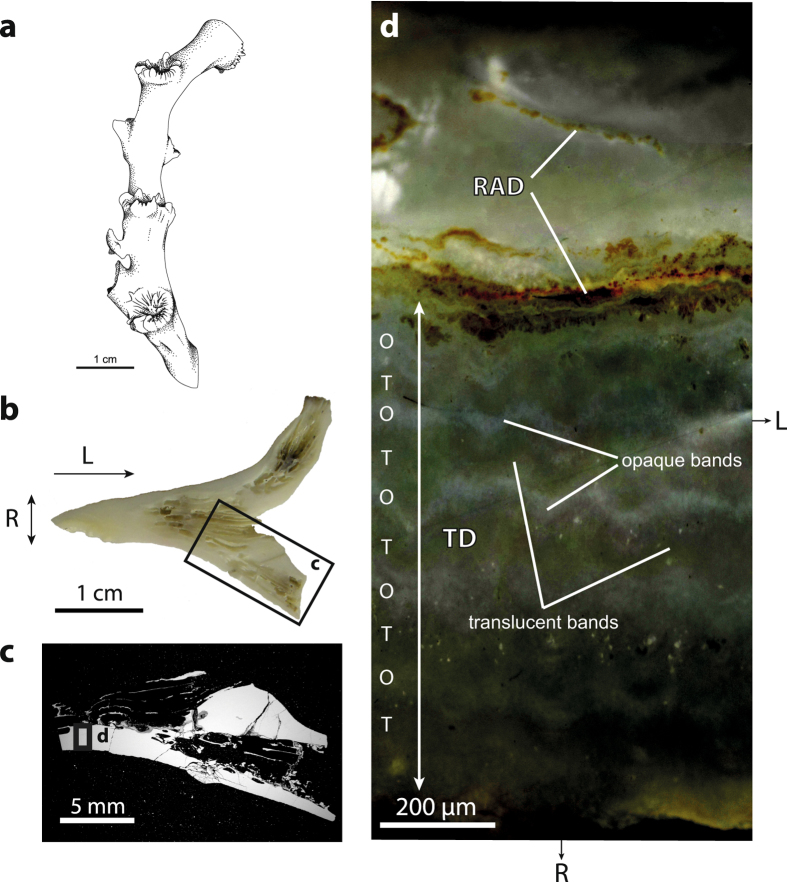
Skeletal architecture of *L. pertusa*. (**a**) Sketch showing a typical branch of *L. pertusa* formed by a succession of corallites. Light (**b**) and electron (**c**) micrographs of the area of investigation. (**d**) Reflected-light image of the area highlighted in (c) displaying a thick rapid accretion deposit (RAD) area running horizontally across the view, and a thinner RAD lamella in the internal part of the corallite wall. Smaller roughly equidimensional RADs (not shown, s. Fig. 2) are also present within the thickening deposit (TD). Alternation of opaque (O) and translucent (T) bands can be distinguished in the TD area. Radial and longitudinal growth directions are indicated by R and L, respectively. Artwork 1a by A. Lethiers, used with permission.

There are considerable uncertainties and gaps in knowledge regarding just how *L. pertusa* constructs its aragonitic skeleton. It is thought that skeletal growth progresses from rapid accretion deposit (RAD) areas^9^, traditionally referred to as centres of calcification^10^. Such biomineralized zones ultimately serve to structurally support the rest of the skeletal features, namely the thickening deposits (TD). Opaque and translucent bands are optically visible in the radial direction of the coral wall^11–13^ (Fig. 1). The question whether alternation of opaque and translucent bands can be correlated with temporal changes in the environment is still debated, thus challenging its use as a sclerochronologic tool^14, 15^.

On the basis of scanning electron microscopy (SEM) observations, *L. pertusa* is considered to construct its skeleton through successive growth phases in a complex three dimensional arrangement^16^. RADs are generally thought to form from the reorganization of amorphous calcium carbonate (ACC) precursors^17–19^, whereas the TD regions are made of elongated aragonite crystals forming the coral wall^16, 18^.

During the last decade, electron backscatter diffraction (EBSD) has proved to be an extremely useful tool to investigate the microstructure of biominerals^20^. Regarding corals, EBSD has been used to study the effects of diagenesis on the structural architecture of *Porites* sp.^21, 22^. The multilevel skeleton organization of the red coral *Corallium rubrum* has been described in detail by Vielzeuf *et al*.^23^ and Floquet and Vielzeuf^24^. More recently, the technique has been used by Coronado *et al*.^25^ to unveil the microstructure of Carboniferous tabulate corals belonging to the Syringoporicae family, and by Hennige *et al*.^8^ to identify skeletal fusion-zones between individual corallites of *L. pertusa*.

We present here the first detailed EBSD study focussing on the microstructure of *L. pertusa* in order to better understand processes leading to the formation and growth of the corallite wall in the RAD and TD areas. A revised explanation of the origin of opaque and translucent bands is given based on textural variation of the aragonite crystals. We critically examine the degree of ordering at a variety of spatial scales and propose a new model for the self-regulation of observed structures. Understanding details of the skeletal architecture of *L. pertusa* provides an insight into the structural resilience of this cold-water coral, and how its possible demise may affect both biodiversity and carbon sequestration in the oceans. Our findings also have a bearing on the interpretation of geochemical proxy data from the coral skeleton used to examine signals of environmental change. Finally, we suggest that EBSD may be used to provide a similar level of insight into biogenic controls on crystallography in other important habitat-forming organisms.

## Results

### Crystal architecture of the skeleton wall

**Figure 2:**
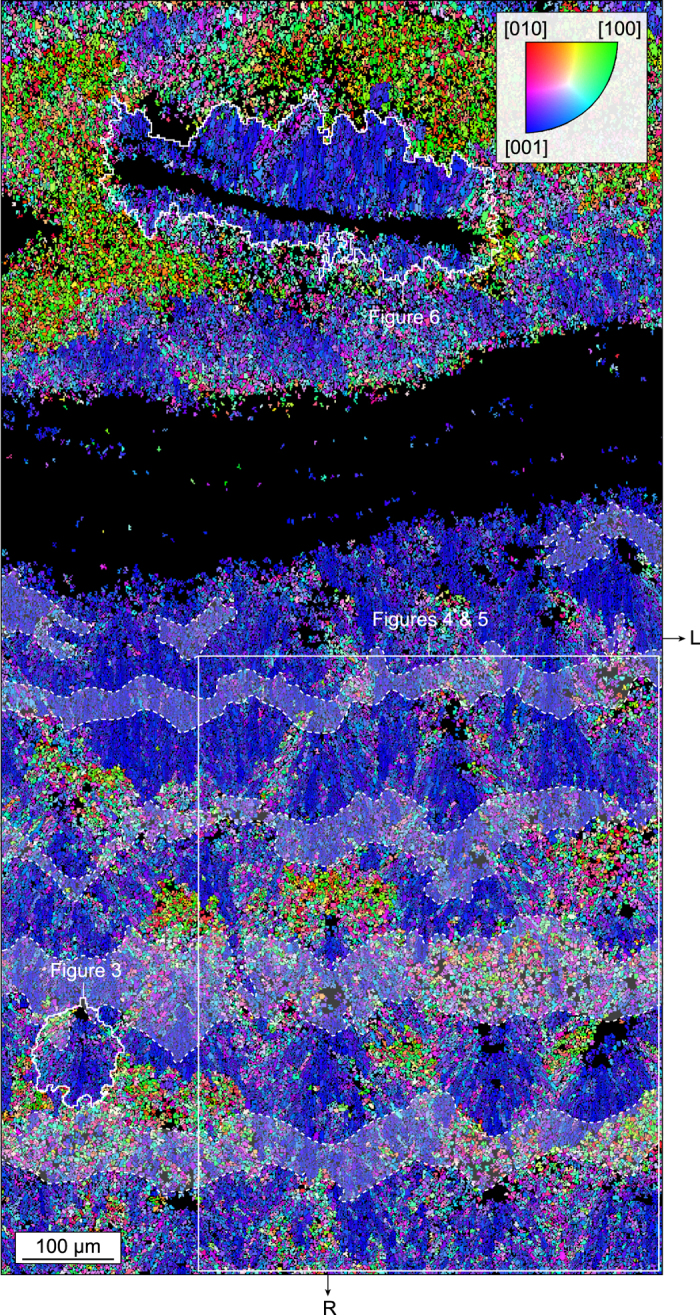
Crystal architecture of the skeleton wall. Inverse pole figure (IPF) map across the corallite wall of *L. pertusa* corresponding to the area displayed in Fig. 1d. The map shows the distribution of aragonite crystallographic axes with respect to the radial growth direction R of the corallite. The dominant bluish colour in the external part of the corallite wall (i.e. the TD area located in the lower half of the map) indicates that aragonite [001] axes are preferentially oriented parallel to R (within a tolerance of ~±45°), i.e. normal to the corallite margin. The crystallites coloured in reddish and greenish are those for which the axes close to R are [010] and [100], respectively, i.e. those intersecting the acquisition surface at high angles. A distinctive feature of the internal part of the corallite wall (upper half of the map) is the RAD lamella from which aragonite needles are radiating along their [001] axes. RADs appear black because they did not produce any diffraction pattern, possibly because of poor crystallinity and/or small crystal size. Optically opaque bands identified in Fig. 1d have been superimposed in light grey and delimited by dashed lines. R and L as in Fig. 1.

The skeleton wall of *L. pertusa* shows different microstructures on each side of the main RAD (Fig. 2). The external part of the corallite wall (corresponding to the TD area) consists of a three-dimensional array of fan-like sclerodermites crystallized from roughly equidimensional RADs. In contrast, the internal part of the corallite wall contains flat RAD lamellae, from which aragonite needles grow without forming fan-like microstructures.

### Crystallographic anatomy of sclerodermites

**Figure 3:**
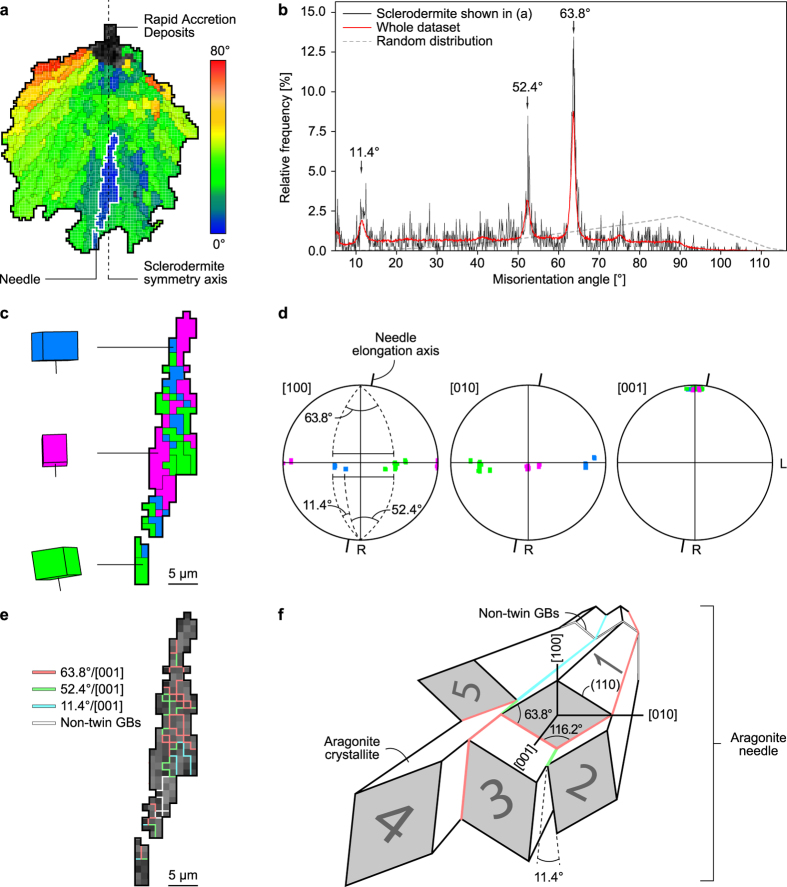
Crystallographic anatomy of sclerodermites. (**a**) EBSD cumulative misorientation map of the sclerodermite outlined in Fig. 2 showing the orientation of aragonite [001] axes with respect to the sclerodermite symmetry axis (dashed line). Only standard (i.e. non-twin) grain boundaries delimiting domains with misorientations >10° are indicated for clarity. The needle outlined in white has been selected for further investigation. (**b**) Diagram showing the non-random distribution of misorientation angles between the adjacent crystallites of the sclerodermite shown in (**a**). High relative frequencies are observed for misorientation angles of 11.4°, 52.4°, and 63.8°. (**c**) Trichromic EBSD map of the aragonite needle outlined in (a) illustrating the approximate orientation of the aragonite unit cell and [001] axes. (**d**) Pole figures of the needle shown in (**c**) displaying the strong clustering of aragonite [001] axes close to the needle elongation axis and the pseudo-hexagonal misorientation of [100] and [010] axes in a plane oriented normal to R. Pole figures are represented on lower hemisphere equal area projections. The radial and longitudinal growth directions of the corallite are indicated by R and L, respectively. Colour code as in (**c**). (**e**) EBSD twin boundary map of the needle shown in (**c**) displaying the misorientation angle/axis pairs between adjacent crystallites. Most of the grain boundaries are twin boundaries delimiting crystallites characterized by misorientations of 11.4°, 52.4°, and 63.8° around [001]. Note that non-twin grain boundaries are located in the bent section of the needle. (**f**) Sketch illustrating the angular and facial relationships between pseudo-hexagonally twinned crystallites within an aragonite needle. Misorientation angles between grains 1 & 2, 2 & 3, and 1 & 5 are 63.8°, 52.4° and 11.4°, respectively. Colour code as in (**e**).

A sclerodermite is typically 100–250 µm long and ~100 µm wide, with a symmetry axis oriented parallel to the radial growth direction (R) of the corallite. It is made of acicular aragonite needles, ~10 µm in diameter and ~50 µm in length, radiating from the RAD along their [001] axes. As a consequence of radiating growth, the angle formed by [001] axes and the sclerodermite symmetry axis increases towards the sclerodermite periphery up to several tens of degrees (Fig. 3a). Each needle contains smaller subunits called crystallites, <5 µm in size, which are slightly elongated along [001]. The distribution of misorientation angles between adjacent crystallites is predominantly non-random, with peaks forming at angles of 11.4°, 52.4°, and 63.8° (Fig. 3b). EBSD data show that crystallites belonging to the same needle are roughly dominated by three main crystallographic orientations distributed around [001] (Fig. 3c). This result can be further refined using the same data represented in pole figures (Fig. 3d), which show a strong clustering of aragonite [001] towards the radial growth direction R of the corallite, and clusters of [100] and [010] axes in a plane oriented normal to R. Misorientation angles of 63.8° are observed between each cluster, whereas secondary maxima of the same cluster have misorientation angles of 11.4° (the subtraction of the two values being equal to 52.4°). The predominance of these misorientation angles along with the observed distribution of crystallographic axes is attributed to pseudo-hexagonal polycyclic twinning of aragonite on {110}, which is typically characterized by rotations of 11.4°, 52.4°, and 63.8° around [001]. As a consequence of polycyclic twinning, most grain boundaries between aragonite crystallites are special twin boundaries (Fig. 3e,f). Non-twin grain boundaries are a minority but, when present, they generally correspond to bent sections of the needles. Bending does not exceed a few degrees and rarely occurs more than once or twice within a needle. This feature is significant enough, however, to account for the slight misorientation observed between the needle elongation axis and the orientation of [001] axes (Fig. 3d).

### RAD distribution and transcurrent layering

**Figure 4:**
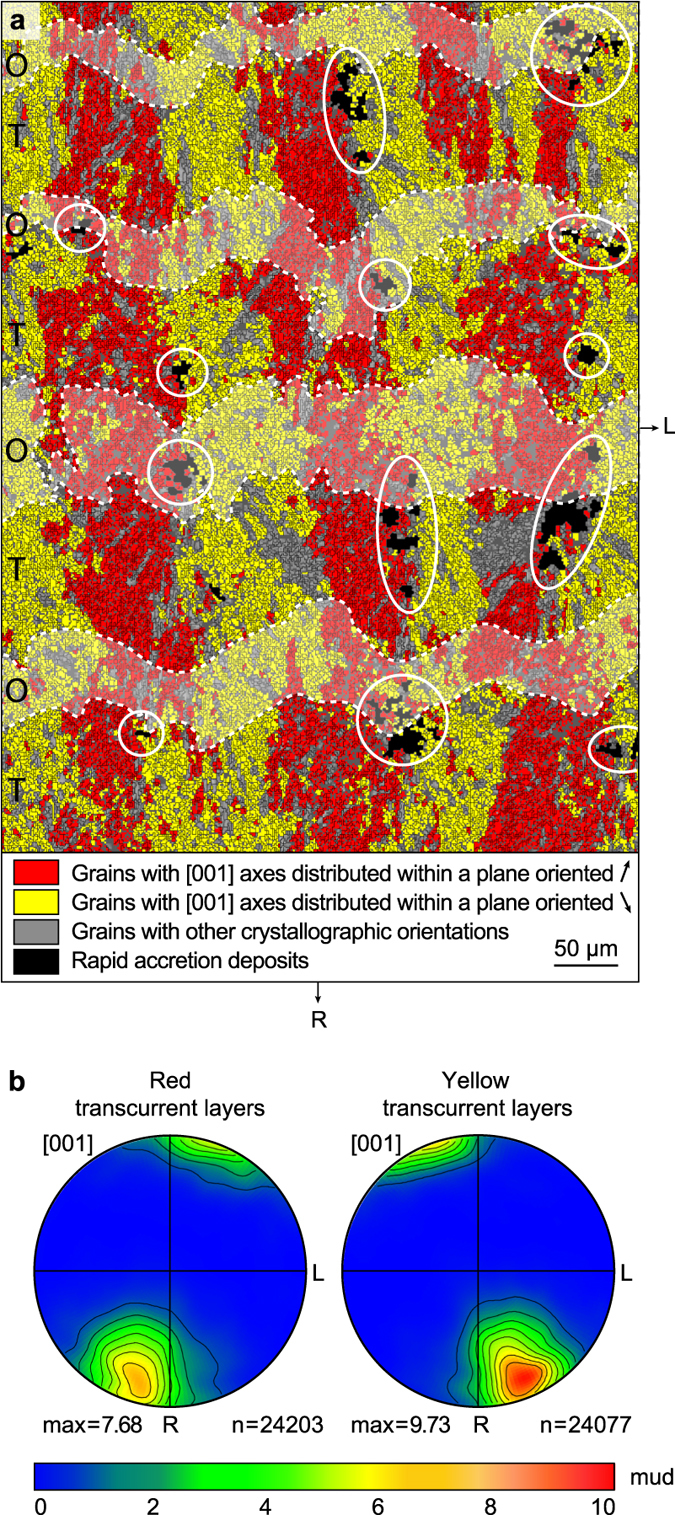
RAD distribution and transcurrent layering. (**a**) Dichromic EBSD map of the area squared in Fig. 2 showing the occurrence of continuous transcurrent layers normal to the corallite wall. Pixels of the same colour have [001] axes oriented within the same reference plane (within a tolerance of ±15°). The reference plane for the red pixels has a strike of N25°E, whereas that for the yellow pixels is N25°W. Both reference planes are normal to the section. RADs are indicated by white ellipses and opaque bands are shown by dashed lines. (**b**) Pole figures of aragonite [001] axes for the left and right transcurrent layers represented on lower hemisphere equal area projections. Contours have been generated using a Gaussian half-width of 12° using only one point per grain to avoid bias caused by possibly non-uniform grain sizes. The total number of grains (n) used for calculation is shown at the bottom right of each plot (adjacent grains were considered distinct for misorientation >5°). The maximum orientation density expressed in multiples of uniform distribution (mud﻿, scale bar) is indicated. R and L as in Figs 2 and 3. Opaque (O) and translucent (T) bands are indicated.

RADs are not distributed randomly within the TD area. They are aligned within planes oriented normal to the corallite wall, preferably at the outer edge of opaque bands. This regular spatial distribution of the RADs results in a remarkable alignment of sclerodermite needles illustrated by a succession of continuous transcurrent layers (colour-coded in yellow and red in Fig. 4a) alternating longitudinally at a right angle to the corallite wall. Pairs of transcurrent layers, which result from the stacking of sclerodermites on top of each other, form the trabeculae described by several authors^17, 26^. Boundary zones between adjacent trabeculae are RAD-free and correspond to areas in which needles impinge at high angles.

Pole figures of Fig. 4b indicate a non-uniform spread of aragonite [001] axes within transcurrent layers. This is illustrated by a strong clustering of axes at an angle of ~25° on either side of the sclerodermite symmetry axis. Interestingly, the clustering strength is significantly larger in the yellow colour-coded than in the red colour-coded transcurrent layers, with multiple of uniform distribution (mud) values of 9.73 for the former and 7.68 for the latter.

### Specific crystallographic misorientations in opaque and translucent bands

**Figure 5:**
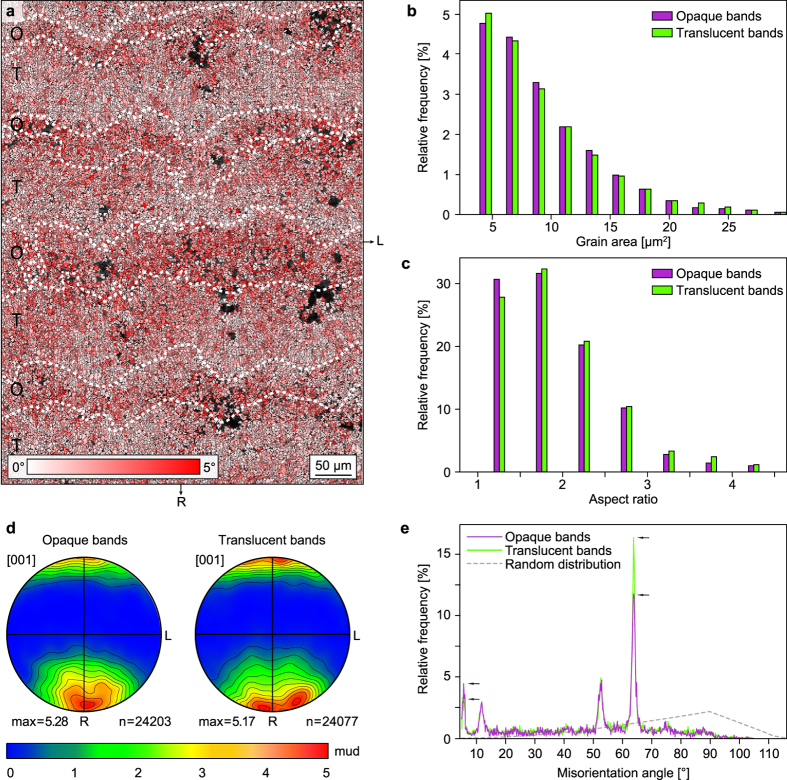
Specific crystallographic misorientations in opaque and translucent bands. (**a**) EBSD local misorientation map of the area squared in Fig. 2 and shown in Fig. 4a. Only misorientations <5° were considered in this map. A pixel is coloured in red when its average misorientation with respect to the 11 × 11 surrounding pixels is close to 5°. A pixel is coloured in white when its average misorientation with respect to the 11 × 11 surrounding pixels is close to 0°. The map shows a higher abundance of red pixels in opaque bands (O) and of white pixels in translucent bands (T). (**b**) Histogram displaying the grain area distribution for opaque and translucent bands. Crystallites represented by a single pixel (2.25 μm^2^) have been discarded for clarity. (**c**) Histogram showing the aspect ratio distribution for opaque and translucent bands. (**d**) Pole figures of aragonite [001] axes showing the crystallographic preferred orientation of [001] axes for opaque and translucent bands. Note that [001] axes in opaque bands have a single crystallographic preferred orientation parallel to R, whereas two maxima are observed in translucent bands. Projection and contouring parameters as in Fig. 4. (**e**) Diagram showing the distribution of misorientation angles between adjacent grains in the opaque and translucent bands. Opaque bands display a lower abundance of misorientation angles of 63.8°, and a higher abundance of misorientations at ~5° (s. arrows), which fits well with the spatial distribution of pixels colour-coded in red in the EBSD map shown in (a).

Opaque and translucent bands show subtle differences in crystallographic preferred orientations and microstructures. When considering misorientations <5°, crystallites located within opaque bands show higher average misorientation angles than those in translucent bands (Fig. 5a). This difference cannot be convincingly attributed to morphological factors, as crystallites from both opaque and translucent bands have similar grain area (Fig. 5b) and aspect ratio (Fig. 5c) distributions. Pole figures generated from either band types are also quite similar (Fig. 5d), with aragonite [001] axes oriented preferentially towards the corallite radial direction R. Careful observation, however, indicates a more dispersed distribution of [001] axes within the RL plane for translucent bands than for opaque ones, with two maxima on each side of the R direction for the former, and only one maximum close to R for the latter (Fig. 5d). This small discrepancy in the distribution of [001] axes can be explained by the more widespread occurrence of well-developed needles in the translucent bands than in the opaque ones (s. Figs. 2 and 4). When looking at the distribution of misorientation angles (Fig. 5e), values of 63.8° are more common in the translucent bands, whereas values of ~5° are predominant in the opaque bands (the distribution of 11.4° and 52.4° being roughly equal). This feature is in agreement with the notion that the sclerodermites are slightly better developed in the translucent bands than in the opaque ones.

### Aragonite growth normal to calcification interface

**Figure 6:**
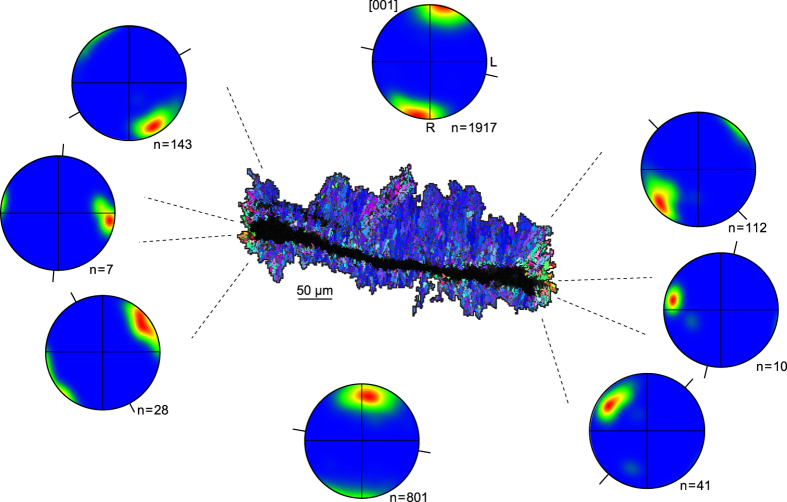
Aragonite growth normal to calcification interface. Inverse pole figure (IPF) map of the crystallization lamella highlighted in Fig. 2, along with aragonite [001] pole figures corresponding to the sectors delimited by dashed lines. Projection and contouring parameters as in Figs 4 and 5, except that the Gaussian half-width has been set to 20° to avoid non-representative peaks in sectors with poor statistics (i.e. small n). The orientation of the calcification interface is indicated by black lines. R and L are shown only for the pole figure at the top centre. IPF map colour code as in Fig. 2.

In the internal part of the crystallite wall RADs take the form of flat lamellae (Fig. 2) from which acicular aragonite needles grow. The pole figures presented in Fig. 6 show a strong clustering of aragonite [001] axes roughly parallel to the needle elongation axis, i.e. at right angle to the calcification interface. This feature is particularly striking at the tips of the lamella, in which [001] axes are systematically distributed normal to the calcification interface despite its abrupt change in orientation.

## Discussion

**Figure 7:**
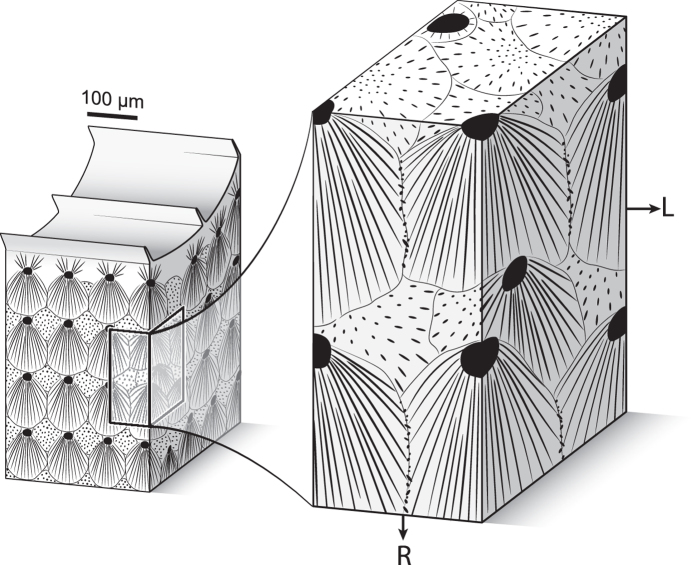
Three-dimensional model of the sclerodermite organization. Black dots and lines represent RADs and needles, respectively. On the right panel, the left part of the front surface corresponds to a yellow colour-coded transcurrent layer (as defined in Fig. 4) while the right part corresponds to a red colour-coded transcurrent layer. Because sclerodermites form three-dimensional bundles expanding towards all free directions, needles growing from RADs lying slightly off-axis with respect to the radial growth direction intersect the acquisition surface at high angles and form the multi-coloured patches visible in Fig. 2. They do not, however, interrupt the continuity of transcurrent layers. R and L as in Fig. 2. Artwork by A. Lethiers, used with permission.

Crystallographic data reveal a remarkably high degree of multi-scale self-ordering in the skeletal microstructure of cold-water coral *L. pertusa*. Evidence for this architectural organization includes the occurrence of ubiquitous polycyclic twinning between needle-forming crystallites, the axially-symmetric arrangement of needles into bundle-shaped sclerodermites, and the formation of crystallographically distinct transcurrent layers normal to the corallite wall. The occurrence of continuous transcurrent layers requires a non-random distribution of RADs within the microstructure. The alignment of RADs within planes with only minimal off-axis shift from the radial direction, together with their preferential positioning on the outer edges of opaque bands and/or yellow colour-coded transcurrent bands with respect to both radial and longitudinal growth directions, lead to a surprisingly regular tridimensional array in which order is only marginally disrupted. In this configuration, aragonite [001] axes are always kept within the reference plane of transcurrent layers, even for the sclerodermite needles intersecting the acquisition surface at high angles (Fig. 7). The highly-ordered nature of the microstructure is further illustrated within opaque and translucent bands, where crystallites display specific abundances of reciprocal crystallographic misorientations. The presence of sclerodermites in both opaque and translucent bands without discrimination or marked stops refutes the accepted idea that optical opacity of bands is caused by strongly disorganized crystallographic orientations.

Crystallographic data also shed new light on the growth mechanisms at play during the skeleton formation of *L. pertusa*. Although sclerodermites form individually from single source regions (i.e. RADs), simultaneous growth of adjacent bundles means that crystal growth competition occurs, as previously suggested by Barnes^27^. The degree of freedom available for needles to grow can be inferred from the distribution of aragonite [001] axes in transcurrent layers. A stronger clustering of axes in yellow colour-coded bands compared to the red colour-coded bands suggests that needles of the former, located on the front edge of mineralization on the longitudinal axis, had more freedom to grow than the latter, which had to compete for space with the needles of the previously-formed adjacent sclerodermites. This observation reinforces the idea of simultaneous radial and longitudinal growth of the corallite wall of *L. pertusa*, and strongly supports coral biomineralization models^9, 28^ and the interpretation of growth increments in the skeleton^14, 16^.

Aragonite needles always grow with [001] axes normal to the calcification interface, would it be from equidimensional or lamellar RAD areas. Such a preferred orientation clearly suggests the prevalence of an interface-controlled growth process in *L. pertusa*. Extra-cellular mucus substances produced by cold-water corals, including *L. pertusa*, have previously been reported to serve several roles such as protection from attacks of endolithic and boring organisms, trapping of food particles and initiation of biomineralization processes^29–31^. Our findings confirm the possible role of extra-cellular mucus as a pre-requisite for the formation of RADs in initiating new aragonite nucleation centres in cold-water corals.

Progressing acidification of the contemporary oceans is predicted to result in shoaling of the aragonite saturation horizon and a reduction in calcification rates^32^. Ocean acidification will also cause a decrease in breaking strength of *L. pertusa* ^8^, which we suggest is most likely to propagate along the structurally weakest zones of the skeleton, namely the RAD, although the effects of this acidification on the degree of self-ordering is currently unconstrained. The highly-ordered structures described for *L. pertusa* likely contribute to the physical robustness of its skeleton, making it resistant to strong hydrodynamic currents. Several studies have reported preferred orientations of cold-water coral colonies, including that of *L. pertusa* ^33^, with a correlation between colony orientation and local hydrodynamic and sediment supply characteristics. The capacity of *L. pertusa* to control its orientation for optimum growth is enhanced by its ability for remarkable multi-scale self-ordering of its skeletal structure.

## Materials and Methods

### Sample preparation

The specimen of *L. pertusa* studied here originated as a coral rubble grab sampled at a water depth of 340 m in the Porcupine Seabight, offshore SW Ireland (51°25′12″N, 11°30′13″W), during the *RV Celtic Explorer* CE-13001 cruise of January 2013. Standard sample preparation involved cleaning at room temperature in an aqueous solution of hydrogen peroxide (H_2_O_2_) 5%, and several rinsing steps in an ultrasonic bath filled with deionised water. The specimen surface was carefully examined before analysis to avoid potential areas of bioerosion or other alteration features.

### Electron backscatter diffraction and scanning electron microscopy imaging

Electron backscatter diffraction (EBSD) is an SEM-based technique which allows for an *in-situ* determination of the lattice orientation within crystalline solids. For each point of analysis, a diffraction pattern (also called EBSD or Kikuchi pattern) is generated through the interaction of the electron beam with the crystal lattice. The pattern is indexed and compared to a structure file containing the crystallographic parameters of the material, from which the orientation of the crystal lattice can be determined. Areas of interest can be scanned and displayed as colour-coded orientation maps, such as misorientation or inverse pole figure (IPF) maps. Points of analysis are characterized by three Euler angles (φ_1_, Φ, φ_2_) which can be compiled into pole figures to display the orientation of crystal axes (e.g. aragonite [001]) with respect to a sample reference frame (e.g. longitudinal and radial growth directions in corals). A review of the basic principles of EBSD can be found in Prior *et al*.^34^.

The portion of the specimen to be investigated using EBSD was sawed longitudinally (Fig. 1), hot-pressed in conductive resin, and polished stepwise down using diamond paste (3 µm for three hours, then 1 µm for three hours) and colloidal silica suspension. No carbon coating was applied to ensure high-quality diffraction patterns. EBSD analyses were carried out at the University of Lausanne, Switzerland, using a Tescan Mira LMU FE-SEM operated at an acceleration voltage of 20 kV, a probe current of 2.5 nA, a working distance of 23 mm, and a sample tilt of 70°. The instrument was equipped with a Nordlys S detector and the AZtec 2.4 software package released by Oxford Instruments. The unit cell parameters for the aragonite structure file were chosen with a[100] = 4.96 Å, b[010] = 7.97 Å, c[001] = 5.74 Å, which corresponds to the definition used by the International Union of Crystallography and the Mineralogical Society of America. As there is no absolute convention in the orthorhombic system about priority for a long or a short unit-cell dimension being along a specific axis (s. e.g. Zou *et al*.^35^, p. 23), care must be taken in the interpretation of aragonite crystallographic data reported in the literature. The calcite structure file taken from the HKL database, with unit cell parameters a[100] = 4.99 Å, b[010] = 4.99 Å, c[001] = 17.06 Å, was used to exclude the potential occurrence of secondary calcite in the microstructure.

EBSD maps were collected using a point collection time of 0.15 s and a step size of 1.5 µm. Points of analysis with mean angular deviation (MAD) >1° were considered unreliable and discarded. Post-processing of the EBSD maps involved noise-reduction using the standard wildspike correction method of the AZtec software and a six-neighbour zero solution extrapolation.

## Electronic supplementary material

Supplementary Figure

## References

[1] Roberts, J. M., Wheeler, A. J., Freiwald, A. & Cairns, S. D. *Coldwater Corals: the Biology and Geology of Deep-Sea Coral Habitats*. Cambridge University Press, Cambridge, 352p. (2009).

[2] Freiwald, A. *et al*. *Cold-water Coral Reefs*. UNEP-WCMC, Cambridge, UK, 84 p. (2004).

[3] Wheeler, A. J. *et al*. Deep-water coral mounds on the Porcupine Bank, Irish Margin: preliminary results from the Polarstern ARK-XIX/3a ROV cruise. In: *Cold-Water Corals and Ecosystems* (eds Freiwald, A. & Roberts, J. M.), pp. 393–402 (Springer, Berlin, 2005).

[4] Etnoyer, P. & Morgan, L. E. Habitat-forming deep-sea corals in the Northeast Pacific Ocean. In: *Cold-Water Corals and Ecosystems* (eds Freiwald, A. & Roberts, J. M.), pp. 331–343 (Springer, Berlin, 2005).

[5] Costello, M. J. *et al*. Role of cold-water *Lophelia pertusa* coral reefs as fish habitat in the NE Atlantic. In: *Cold-Water Corals and Ecosystems* (eds Freiwald, A. & Roberts, J. M.), pp. 771–805 (Springer, Berlin, 2005).

[6] Titschack J (2016). Mediterranean cold-water corals – an important regional carbonate factory?. The Depositional Record.

[7] Zheng M-D, Cao L (2014). Simulation of global ocean acidification and chemical habitats of shallow- and cold-water coral reefs. Advances in Climate Change Research.

[8] Hennige SJ (2014). Self-recognition in corals facilitates deep-sea habitat engineering. Sci. Rep..

[9] Stolarski J (2003). Three-dimensional micro- and nanostructural characteristics of the scleractinian coral skeleton: a biocalcification proxy. Acta Palaeontol. Pol..

[10] Ogilvie MM (1896). Systematic study of Madreporan corals. Philos. T. R. Soc. Lond..

[11] Wainwright SA (1964). Studies of the mineral phase of coral skeleton. Exp. Cell Res..

[12] Lazier AV, Smith JE, Risk MJ, Schwarcz HP (1999). The skeletal structure of *Desmophyllum cristagalli*: the use of deep-water corals in sclerochronology. Lethaia.

[13] Risk, M. J., Hall-Spencer, J. & Williams, B. Climate records from the Faroe-Shetland Channel using *Lophelia pertusa* (Linnaeus, 1758). In: *Cold-Water Corals and Ecosystems* (eds Freiwald, A. & Roberts, J. M.), pp. 1097–1108 (Springer, Berlin, 2005).

[14] Gass SE, Roberts JM (2011). Growth and branching patterns of *Lophelia pertusa* (Scleractinia) from the North Sea. J. Mar. Biol. Assoc. U.K..

[15] Knutson DW, Buddemeier RW, Smith SV (1972). Coral chronometers - seasonal growth bands in corals. Science.

[16] Mouchi V (2014). Potential seasonal calibration for palaeoenvironmental reconstruction using skeletal microstructures and strontium measurements from the cold-water coral *Lophelia pertusa*. J. Quaternary Sci..

[17] Cohen, A. L. & McConnaughey, T. A. Geochemical perspectives on coral mineralization. In: *Biomineralization* (eds Dove, P. M., De Yoreo, J. J. & Weiner, S.). *Rev. Mineral. Geochem*. **54**, 151–187 (2003).

[18] Rollion-Bard C, Blamart D, Cuif J-P, Dauphin Y (2010). *In situ* measurements of oxygen isotopic composition in deep-sea coral, *Lophelia pertusa*: Re-examination of the current geochemical models of biomineralization. Geochim. Cosmochim. Ac..

[19] Falini G (2013). Control of aragonite deposition in colonial corals by intra-skeletal macromolecules. J. Struct. Biol..

[20] Cusack M (2016). Biomineral electron backscatter diffraction for palaeontology. Palaeontology.

[21] Cusack M (2008). Electron backscatter diffraction (EBSD) as a tool for detection of coral diagenesis. Coral Reefs.

[22] Dalbeck P (2011). Identification and composition of secondary meniscus calcite in fossil corals and the effect on predicted sea surface temperature. Chem. Geol..

[23] Vielzeuf D (2010). Multilevel modular mesocrystalline organization in red corals. Am. Mineral..

[24] Floquet N, Vielzeuf D (2011). Mesoscale twinning and crystallographic registers in biominerals. Am. Mineral..

[25] Coronado I, Pérez-Huerta A, Rodriguez S (2015). Crystallographic orientations of structural elements in skeletons of Syringoporicae (tabulate corals, Carboniferous): Implications for bomineralization processes in Palaeozoic corals. Palaeontology.

[26] James NP (1974). Diagenesis of Scleractinian corals in the subaerial vadose environment. J. Paleo..

[27] Barnes DJ (1970). Coral skeletons: An explanation of their growth and structure. Science.

[28] Cuif, J.-P., Dauphin, Y. & Sorauf, J. *Biominerals and fossils through time*. Cambridge University Press, Cambridge, 490 p (2011).

[29] Mortensen PB (2001). Aquarium observations on the deep-water coral *Lophelia pertusa* (L., 1758) (scleractinia) and selected associated invertebrates. Ophelia.

[30] Reitner, J. Calcifying extracellular mucus substances (EMS) of *Madrepora oculata* – a first geobiological approach. In: *Cold-Water Corals and Ecosystems* (eds Freiwald, A. & Roberts, J.M.), pp. 731–744 (Springer, Berlin, 2005).

[31] Zetsche E-M, Baussant T, Meysman FJR, van Oevelen D (2016). Direct visualization of mucus production by the cold-water coral *Lophelia pertusa* with digital holographic microscopy. PLoS ONE.

[32] Roberts JM, Cairns SD (2014). Cold-water corals in a changing ocean. Curr. Opin. Env. Sust..

[33] Gori A (2013). Bathymetrical distribution and size structure of cold-water coral populations in the Cap de Creus and Lacaze-Duthiers canyons (northwestern Mediterranean). Biogeosciences.

[34] Prior DJ (1999). The application of electron backscatter diffraction and orientation contrast imaging in the SEM to textural problems in rocks. Am. Mineral..

[35] Zou, X., Hovmöller, S. & Oleynikov, P. *Electron Crystallography - Electron Microscopy and Electron Diffraction*. Oxford University Press, 344 p (2011).

