# Supplementary material for: Multi-scale crystallographic ordering in the cold-water coral Lophelia pertusa
Source: Sci Rep. 2017 Aug 21;7:8987. doi: 10.1038/s41598-017-09344-5 (PMC5566427; doi:10.1038/s41598-017-09344-5)

« Multi-scale crystallographic ordering in the cold-water coral *Lophelia pertusa* », by Mouchi V., Vonlanthen P., Verrecchia E.P., Crowley Q.G.

Supplementary Figure 1: Scanning electron microscope image of the studied *Lophelia pertusa* specimen. After EBSD analysis, the section was etched with formic acid 2% for 50 seconds and carbon coated. Secondary electron images were taken at the Université Pierre et Marie Curie, Paris, France, using a Zeiss Supra 55VP FE-SEM operated at an acceleration voltage of 3 kV, a probe current of 0.275 nA, and a working distance of 3.3 mm. Orientations of the aragonite needles on this figure correspond to the EBSD results.

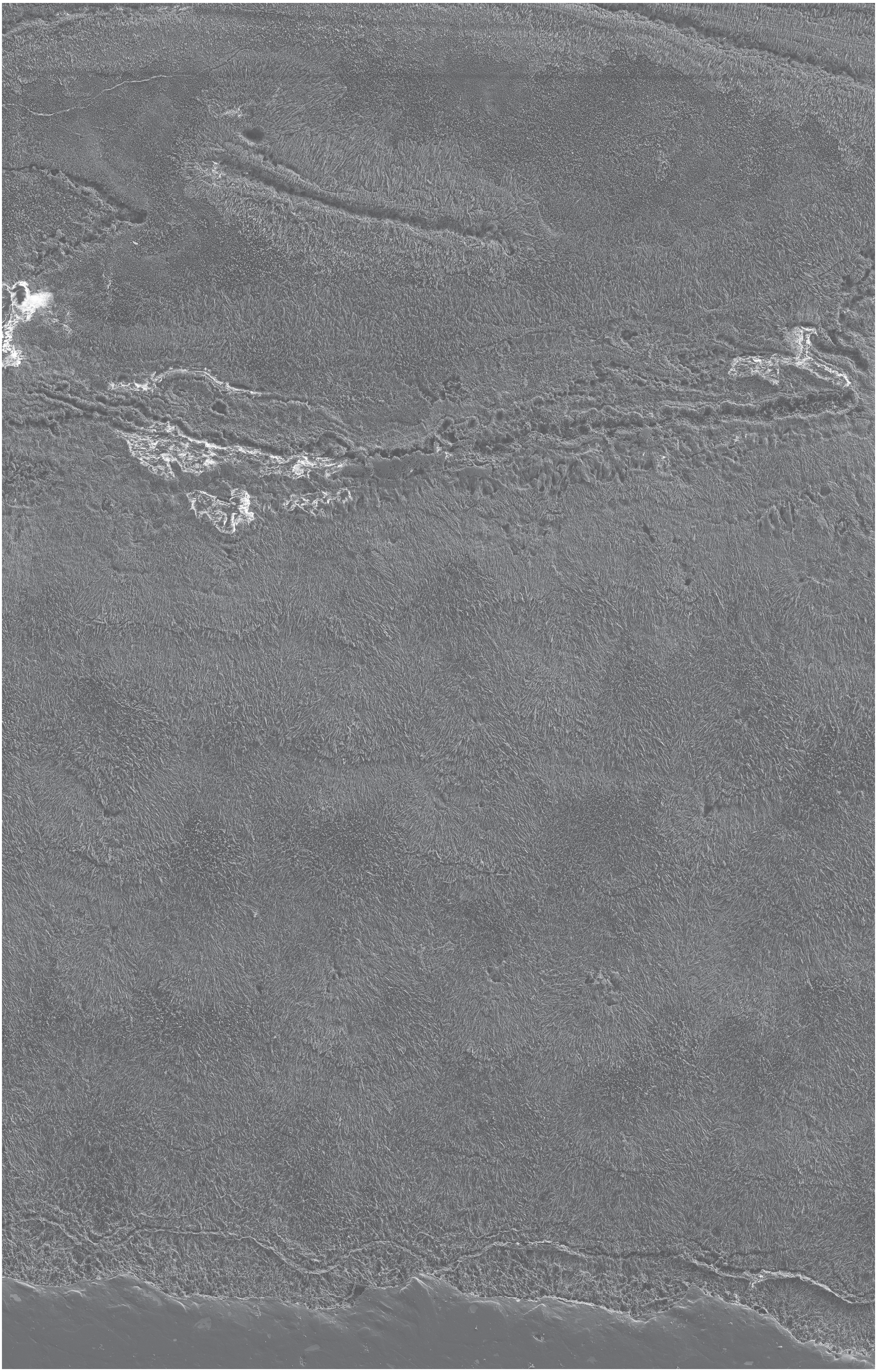

Supplement: Supplementary file 1 — Supplementary Figure [file 41598_2017_9344_MOESM1_ESM.pdf]
